# The hidden side of diversity: Effects of imperfect detection on multiple dimensions of biodiversity

**DOI:** 10.1002/ece3.7995

**Published:** 2021-08-10

**Authors:** Aline Richter, Gabriel Nakamura, Cristiano Agra Iserhard, Leandro da Silva Duarte

**Affiliations:** ^1^ Departamento de Ecologia Universidade Federal do Rio Grande do Sul Porto Alegre Brazil; ^2^ Departamento de Biologia Universidade Federal do Ceará Fortaleza Brazil; ^3^ Departamento de Ecologia Zoologia e Genética Universidade Federal de Pelotas Pelotas Brazil

**Keywords:** assemblage structure, community hierarchical models, detection probability, environmental gradients, fruit‐feeding butterflies, functional traits, phylogenetic diversity

## Abstract

1. Studies on ecological communities often address patterns of species distribution and abundance, but few consider uncertainty in counts of both species and individuals when computing diversity measures.
2. We evaluated the extent to which imperfect detection may influence patterns of taxonomic, functional, and phylogenetic diversity in ecological communities.
3. We estimated the true abundance of fruit‐feeding butterflies sampled in canopy and understory strata in a subtropical forest. We compared the diversity values calculated by observed and estimated abundance data through the hidden diversity framework. This framework evaluates the deviation of observed diversity when compared with diversities derived from estimated true abundances and whether such deviation represents a bias or a noise in the observed diversity pattern.
4. The hidden diversity values differed between strata for all diversity measures, except for functional richness. The taxonomic measure was the only one where we observed an inversion of the most diverse stratum when imperfect detection was included. Regarding phylogenetic and functional measures, the strata showed distinct responses to imperfect detection, despite the tendency to overestimate observed diversity. While the understory showed noise for the phylogenetic measure, since the observed pattern was maintained, the canopy had biased diversity for the functional metric. This bias occurred since no significant differences were found between strata for observed diversity, but rather for estimated diversity, with the canopy being more clustered.
5. We demonstrate that ignore imperfect detection may lead to unrealistic estimates of diversity and hence to erroneous interpretations of patterns and processes that structure biological communities. For fruit‐feeding butterflies, according to their phylogenetic position or functional traits, the undetected individuals triggered different responses in the relationship of the diversity measures to the environmental factor. This highlights the importance to evaluate and include the uncertainty in species detectability before calculating biodiversity measures to describe communities.

## Graphical abstract

Incorporating detection probability in diversity metrics is paramount for reliable estimate of diversity. The effect of imperfect detection could be uneven along the environmental gradient, being a source of bias in the interpretation of ecological patterns. We propose a framework to estimate the effects of imperfect detection for different facets of diversity, the hidden diversity. HD framework evaluates the deviation of observed diversity when compared with diversities derived from estimated true abundances and whether such deviation represents a bias or a noise in the observed diversity pattern.

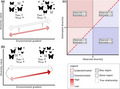

## 1 INTRODUCTION

Estimating the whole biodiversity in a community is a key challenge for ecologists. First, we do not have time and resources to sample all species and individuals that are present in a community. Second, even focusing on a target group, there are large proportions of species or individuals that remain “hidden” (Chao et al., 2017; Devarajan et al., 2020; Guillera‐Arroita et al., 2019; Yoccoz et al., 2001). This occurs since both species and individuals are not perfectly observed in the field (i.e., they are undetected during sampling), and different species have distinct probabilities of being detected (Boulinier et al., 1998; Ribeiro et al., 2016). Classical community analyses commonly ignore imperfect detection, for both incidence and abundance‐based approaches, as well as its effects on diversity measures (DeVries et al., 2012; Pillar & Duarte, 2010; Wiens & Donoghue, 2004). Identify the effects of imperfect detection in classical diversity measures might improve our understanding of relationships between diversity and environmental gradients (Roth et al., 2018) and ultimately the processes that structure the biological communities (Dorazio et al., 2015).

**FIGURE 1:**
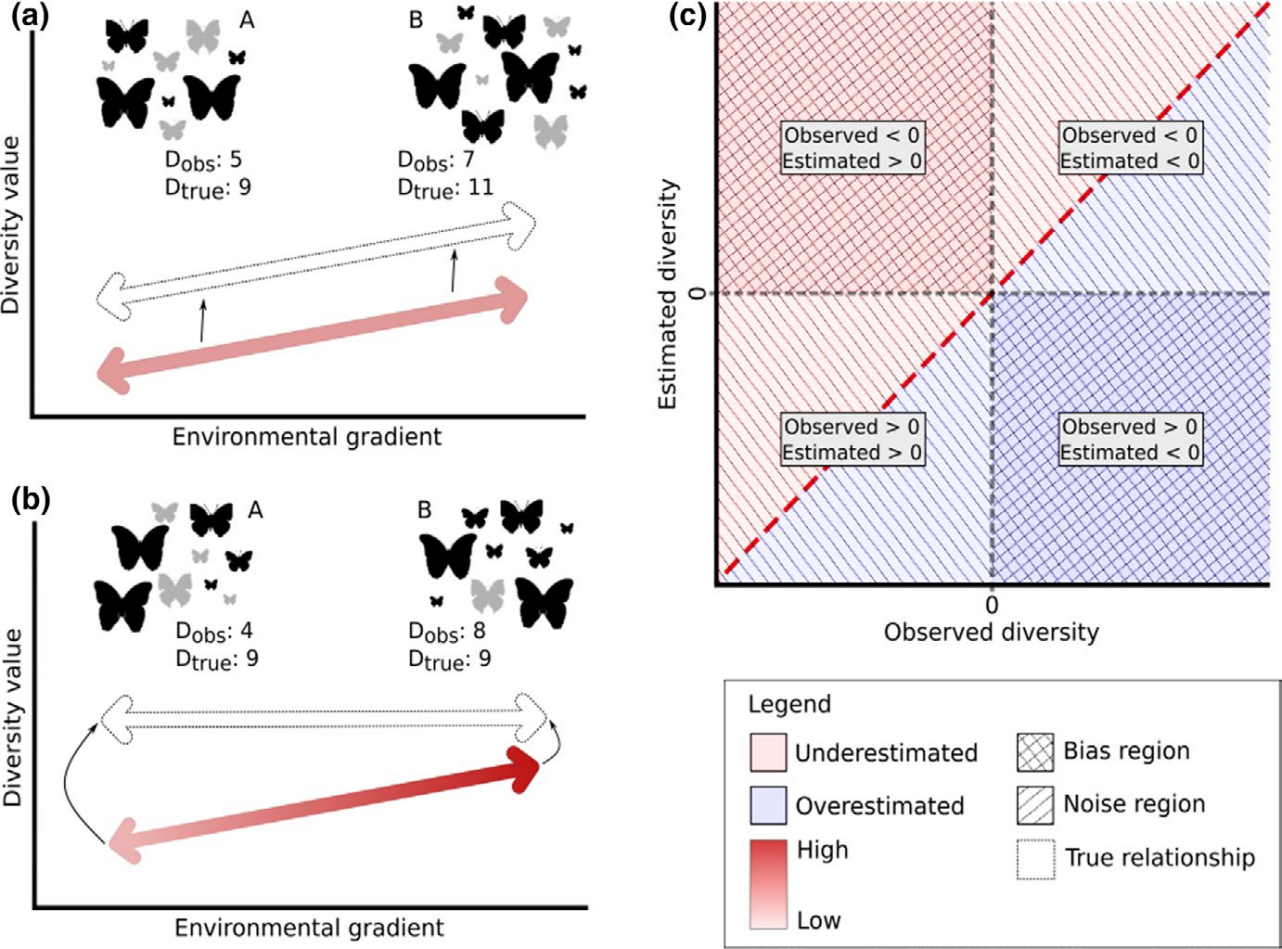
Schematic representations of the hidden diversity framework, demonstrating how imperfect detection can influence the relationship between an environmental factor and a diversity descriptor. Suppose that each set of butterflies represents a community, called A and B. *D*_obs_ is the value of a given diversity measure calculated from an observed community (dark butterflies), which has imperfectly detected species (gray butterflies, probability of detection (*p*) < 1). *D*_true_ represents the real value of this diversity if all species in the community were sampled (*p* = 1). For the sake of simplicity, we will call the difference between the true and observed values hidden diversity (HD). Note that in (a) despite the 4 units decrease in diversity for both communities (HD_A_ = HD_B_ = −4), B remained more diverse than A, and the error associated with imperfect detection was constant along the environmental gradient (red arrow). On the other hand, in (b) the detection probability is not equal along the gradient, which might lead to a bias in the observed relationship between diversity measures and environmental factors, once that *D*_true_ is the same for A and B, but when only observed data are employed to calculate diversity, B is more diverse than A (HD_A_ = −5, HD_B_ = −1). In (c), we show a way to interpret the hidden diversity, which takes into account the signal of the observed and estimated diversity value. The blue and red colors are associated with positive and negative values of HD, respectively. If the observed value (x‐axis) is positive and the estimated value (y‐axis) is negative, we have an overestimation in the diversity value, while if the observed value is negative and the estimated value is positive, we have an underestimation in the diversity, and both are in the critical bias region. When the observed and estimated values have the same sign, the observed patterns tend to hold despite the noise added by imperfect detection

A considerable portion of community studies that employed models that account for imperfect detection (e.g., multispecies hierarchical models) are interested in evaluating the true occurrence or abundance, aiming to guide management practices (Ruiz‐Gutiérrez et al., 2010; Yamaura et al., 2012; Zipkin et al., 2010). But, the effects of imperfect detection are not restricted only to the taxonomic aspect of diversity (e.g., species richness), and our ability in detecting biodiversity patterns may differ among different components of diversity (Iknayan et al., 2014; Jarzyna & Jetz, 2016). Species co‐occurring in communities exhibit different levels of shared evolutionary history and variation in phenotypic traits. These features of species are widely used to infer historical and/or ecological mechanisms determining community assembly patterns (Duarte et al., 2018; Graham & Fine, 2008; Webb et al., 2002). Despite the increase in studies that quantified phylogenetic or functional diversity (de Bello et al., 2015; Tucker et al., 2017), few consider the imperfect detection in species count for calculate it (Chao et al., 2017; Frishkoff et al., 2017) or have quantified the role and magnitude of the effects of imperfect detection on distinct facets of diversity (Jarzyna & Jetz, 2016; Si et al., 2018). If undetected species have unique phylogenetic information or functional traits, by underestimating their contribution to diversity estimate, we are neglecting an ecologically important part of the assemblages (Jarzyna & Jetz, 2016). Consequently, we would observe a more clustered assemblage than they really are (Si et al., 2018). The opposite can also occur when undetected species are phylogenetically or functionally redundant (Jarzyna & Jetz, 2016), and the observed assemblages will overestimate phylogenetic and functional diversity. Furthermore, the detection of species can be biased at some part of the environmental gradient evaluated (Roth et al., 2018). If this occurs, not only the observed diversity pattern can be affected, but also our interpretation of the relationship among diversity and environmental gradients (Figure 1a,b).

Insects are the most species‐rich taxa in the world, which poses a major challenge for ecologists interested in evaluating insect diversity patterns (Thomas, 2005). Among insect groups, butterflies are considered important biological indicators due to their short life cycle and high sensibility to changes in environmental features (Brown & Freitas, 2000; New, 1997). Fruit‐feeding butterflies are a conspicuous guild of tropical butterflies that feed on rotting fruit, carrion, or plant exudates (DeVries, 1988) and represent about 50%–75% of nymphalid diversity in the Neotropical region (Brown, 2005). Assemblages of fruit‐feeding butterflies show high vertical stratification (Devries, 1988; DeVries et al., 2012; Ribeiro & Freitas, 2012; Santos et al., 2017), with the canopy generally being taxonomically more diverse than understory. These strata exhibit a large difference in their microclimatic conditions and habitat structure and hence in their taxonomic composition (Araujo et al., 2020; DeVries et al., 2012; Santos et al., 2017). Whereas Charaxinae, Biblidinae, and Nymphalinae are recognized as canopy‐dwellers, Satyrinae is generally associated with understory sites (Schulze et al., 2001). In a phylogenetic or functional perspective, the composition of those strata could be also dissimilar, once that lineages of fruit‐feeding butterflies exhibit habitat preferences (Fordyce & DeVries, 2016) and individuals tend to show traits that varying according to habitat characteristics and preferences (Graça et al., 2017).

Due to their feeding habit, these butterflies can be sampled with passive and standardized methodologies such as bait traps (Freitas et al., 2021). Unlike other methods to sample butterflies (entomological nets or transect counts), bait traps avoid bias related to variation in the observer or personal expertise about species detection (Boulinier et al., 1998; Kéry & Plattner, 2007; Ribeiro et al., 2016). However, the detection of individuals might be biased by bait attractiveness in different habitats and by the individual ability to find the trap. Weather conditions as wind speed, rain, and temperature can influence the bait volatiles, leading to decreased attractiveness, especially in open habitats (Marini‐Filho & Martins, 2010). Fruit‐feeding butterflies typically use odor cues to locate food, and some groups, such as Charaxinae, can find more accurately their preferred food (Molleman et al., 2005). Further, individuals that have high mobility may often be undetected in a sampling site because it is visiting other sites within their home range (Joseph et al., 2009). Therefore, bearing in mind the intrinsic challenges of sampling in the canopy together with the characteristics of individuals that inhabit this stratum, it is more likely that the canopy has a higher number of undetected individuals than understory, yielding a bias in diversity measured in this stratum.

In this study, we aimed to analyze the extent to which imperfect detection, assessed by the estimates of the true abundance of species, can lead to changes in observed patterns of taxonomic, functional, and phylogenetic diversities of butterflies living in different forest strata (canopy vs. understory). We expect that (a) canopy will show lower individual detection than understory, leading to a source of bias that hides the true diversity value for this stratum. Consequently, this bias induces an erroneous inference when we compare diversity values between canopy and understory. (b) The effect of imperfect detection will be lower for phylogenetic and functional measures concerning taxonomic diversity. In this case, an increment in species number will not be followed by an increment in both phylogenetic and functional diversity, indicating that undetected species are redundant with species sampled in the observed community.

## 2 MATERIALS AND METHODS

### 2.1 Study sites and sampling procedures

The study site was located in Floresta Nacional de São Francisco de Paula (FLONA‐SFP; centered at 29°25′22″S, 50°23′11″W) in southern Brazil. FLONA‐SFP comprises an area of 1,615 ha in the Atlantic Forest biome and is composed of Mixed Ombrophilous Forest with the presence of *Araucaria angustifolia* (Bertol.) Kuntze, as well as patches with *Pinus* sp. and *Eucalyptus* sp. plantations (ICMBio, 2020). The climate of the region is temperate without a dry season, with average annual precipitation close to 2,000 mm and an average annual temperature of 14.5℃ (Sonego et al., 2007).

Fruit‐feeding butterfly assemblages were sampled between November 2016 and March 2017, which correspond to the summer season in the Southern Hemisphere and which is the best period of the year for sampling butterflies in the Atlantic Forest (Iserhard et al., 2017). We adopted standardized methods for sampling fruit‐feeding butterflies in the Neotropical region (Freitas et al., 2014), which consisted in install five traps per sampling unit, which were baited with a mixture of mashed banana and sugarcane juice (Freitas et al., 2021). We performed monthly surveys at six sites of native forest within FLONA‐SFP for 5 months. In each month, the traps remained open for eight to ten consecutive days and every 48 hr the traps were checked and the bait was replaced. This totalizes a sampling effort of 2,520 trap‐days (10 traps ×6 sampling units ×42 sampling days). In each site, we sampled the assemblages of fruit‐feeding butterflies in the canopy (~15 m above the ground, inside canopy tree crowns) and in the understory (1.5 m above the ground) and each stratum was considered as one independent sampling unit. In every trap checking, we measured the temperature of the base of each trap using an infrared thermometer (GM‐300, Benetech^®^).

### 2.2 Community model for abundance data

We employed a modification of the Dorazio‐Royle‐Yamaura model (DRY) (Kéry & Royle, 2016; Yamaura et al., 2011, 2016) to estimate uncertainties in the individual counts for fruit‐feeding butterflies. The modifications allow the model to estimate the mean abundance (*λ_ik_*) and detection probability (*p_ijk_*) for each stratum (Zipkin et al., 2010). We assumed that local abundance remained unchanged during the survey (i.e., closure assumption, Kéry et al., 2005) since we sampled in a narrow time window and that mean abundance and detection probability were independent among species. Abundance for each species *k* at each site *i* is a latent variable (i.e., imperfectly observed) called *N_ik_*, which follows a Poisson distribution:

$$N_{\mathrm{ik}}∼\text{Poisson}\left(\lambda_{\mathrm{ik}}\right)$$

where *λ_ik_* is the mean or expected abundance. We assumed that *λ_ik_* varied among sites depending on species random effects and if point *i* was in the canopy (Strata = 0) or the understory (Strata = 1), thus allowing species‐level effects to differ between the two strata (Zipkin et al., 2010). We also included a slope for the mean temperature obtained from the base of the traps of each site *i* (Temp) and add two random site effects, because samplings were repeated in time (sampling months, SM) for each sampling units (SU), and hence, their measures are not independent within them. We fit the model for biological process using a log‐link function, as follows:

$$\log\left(\lambda_{\mathrm{ik}}\right)=\beta .{\text{can}}_{k}\times \left(1‐{\text{Strata}}_{i}\right)+\beta .{\text{und}}_{k}\times {\text{Strata}}_{i}+\beta 1_{k}\times {\text{Temp}}_{i}+s_{{\text{SU}}_{i}k}+m_{{\text{SM}}_{i}k}$$

where *β*.can and *β*.und are the species‐specific intercepts for canopy and understory, respectively, *β*1 is the species‐specific slope for the temperature effect, and *s* and *m* are the random effects for six sampling units and five sampling months.

We describe the detection process as:

$$y_{\mathrm{ijk}}∼\text{Binomial}\left(N_{\mathrm{ik}},p_{\mathrm{ijk}}\right)$$

where the number of detected individuals *y_ijk_* during visit *j* was obtained with *N_ik_* trials and a probability of successful detection *p_ijk_*. The detection history *y_ijk_* > 0 indicates that the species *k* (1, 2, …, 35) was observed in site *i* (1, 2, …, 12) during the sampling occasion *j* (1, 2, …, 5), while *y_ijk_* = 0 implies the species was undetected. We modeled detectability as a logit‐linear combination of species‐specific detection probabilities dependent on the stratum and two covariates:

$$\text{logit}\left(p_{\mathrm{ijk}}\right)=\alpha .{\text{can}}_{k}\times \left(1‐{\text{Strata}}_{i}\right)+\alpha .{\text{und}}_{k}\times {\text{Strata}}_{i}+\alpha 1_{k}\times {\text{Date}}_{\mathrm{ij}}+\alpha 2_{k}\times {\text{Temp}}_{\mathrm{ij}}$$

where *α*.can and *α*.und are the species‐specific intercepts for canopy and understory, respectively, and *α*1 is the linear effects of the sampling day (transformed to Julian date) and *α*2 is the linear effects of the temperature by day.

All covariates for the biological and observation process were standardized before performing the Bayesian model. The effect of predictors was corroborated whenever 95% of the credible interval (CRI) did not overlap with zero. We defined species‐specific parameters for each stratum and for covariates as coming from normal hyperdistributions, for example, *β*.can*_k_* ~ Normal (*µ_β_*._can_, *τ_β_*._can_), being that these priors describe the heterogeneity among species. We determined vague priors for the hyperparameters that define the mean (*µ*) and precision (*τ*) at the community level, such that *µ* ~ Normal (0, 0.001) and *τ*, that is the inverse of variance (*τ* = sd^−2^), where sd ~ Uniform (0, 10), and these hyperparameters are shared by all species in each stratum (Yamaura et al., 2016). Considering that the mean detection probability must vary between 0 and 1, we defined *µ*_α_ = logit(*µ_α_*._pre_), when *µ_α_*_.pre_ ~ Uniform (0, 1), and then, *α_k_* ~ Normal (*µ_α_*, *τ_α_*). The model was run using the package *jagsUI* (v. 1.4.9, Kellner, 2021) with three Markov Chains Monte Carlo (MCMC), 150,000 iterations with the first 50,000 iterations discarded, and a thinning rate of 100. The model code is given in Appendix S1 (N‐mixture model). These settings of MCMC result in a posterior sampling with 3,000 iterations. We also defined initial values for parameter N and monitored the community mean and species‐level parameters. We checked the convergence of MCMC by R‐hat statistics (Gelman & Rubin, 1992) and graphical visualization.

In addition, we checked and validated the N‐mixture model through simulation of metacommunities (Appendix S2—model validation). For each simulation, we set the mean expected abundance for canopy and understory (*β_s_*_1_ and *β_s_*_2_) or the mean probability for canopy and understory (*α_s_*_1_ and *α_s_*_2_) to vary, while all other parameters were kept constant. For each parameter, we defined true mean values, which we consider low, intermediate, and high, resulting in 12 simulated metacommunities (hereafter treated as setting code A to L). The output of the simulation provided two main information: the true abundance of species for each community (*N_s_*) and the imperfect observed community (yobs*_s_*). The yobs*_s_* was then subjected to the N‐mixture model, and we monitored all parameters estimated. For the biological model, all true values of parameters and hyperparameters fall within 95% of the credible interval of the posterior distribution (Appendix S2—Figures B1–B3), indicating that model was able to recovery true parameters values.

### 2.3 Phylogenetic and functional data

We collected at least one specimen of each butterfly species captured in bait traps for subsequent measurement of functional traits. We selected 12 functional traits to characterize functional diversity in each community, including traits related to flight performance, habitat use, and ecological behavior (Table 1) (Chai & Srygley, 1990; Dudley, 2002; Spaniol et al., 2019). Using the recently proposed phylogeny of Chazot et al. (2019) for Nymphalidae, we obtained the phylogenetic relationships among the 35 species of fruit‐feeding butterflies recorded in this study. We pruned the complete tree to calculate measures of phylogenetic diversity and structure of communities. We used the packages *ape* (v. 5.3, Paradis & Schliep, 2019), and *phytools* (v. 0.6‐44, Revell, 2012) to prune the tree.

**TABLE 1:** Description for the functional traits measured for fruit‐feeding butterflies sampled at FLONA‐SFP, southern Brazil

	Trait name	Type	Measure	Description	References
FWL	Forewing length (mm)	C	Forewing base to apex	Used as a proxy for body size and related with dispersion capacity	Chai and Srygley (1990), Sekar (2012)
TM:TDM	Thorax mass to total body mass ratio	C	The ratio between thorax mass and total body mass	The proportion that represents the investment in thorax mass; related to flight capacity due that thorax allocates the flight muscles	Chai and Srygley (1990)
AM:TDM	Abdomen mass to total body mass ratio	C	The ratio between abdomen mass and total body mass	The proportion that represents the investment in abdomen mass; related to investment in reproductive tissues	Srygley and Chai (1990)
FEA	Functional eye area (mm²)	C	Set of linear eye measurements	Represent the functional visual field; associated with habitat perception	Rutowski (2000), Turlure et al. (2016)
WL	Wing loading (N/m²)	C	Amount of body mass sustained by wing area unit	Related with flight speed and agility and can be associated with adaptative response to environmental gradients	Chai and Srygley (1990), Berwaerts et al. (2002), Turlure et al. (2016)
AR	Aspect ratio	C	The ratio between forewing span squared to forewing area	Express the wing shape; related to flight speed and agility	Chai and Srygley (1990), Berwaerts et al. (2002)
FS	Food specialization	C	Amount of host plants used by immature stages	Express the food habit; lower values represent specialist species, and higher values represent more generalist species.	Graça et al. (2017)
Iridescence	Wing iridescence	B	Presence or absence of iridescence coloration	Related with intra and interspecific visual recognition	Pinheiro et al. (2016), Spaniol et al. (2019)
Eyespots	Wing eyespot	B	Presence or absence of wing eyespots	Related with defense strategies to avoid or deflect attacks of visually hunting predators	Stevens (2005), Olofsson et al. (2010)
Rings	Mimetic ring	B	Member or not of mimetic rings complex	Indicate if species are a member of mimetic rings; related to Mullerian, Batesian or scape mimetic rings	Su et al. (2015), Spaniol et al. (2019)
Camouflage	Camouflage strategies	B	Colorations and shapes that resemble background or environmental structures	Related to capacity to avoid predators	Ruxton et al. (2004), Skelhorn et al. (2010)
Disruptive	Disruptive coloration	B	Conspicuous colorations in the wing's periphery that disguises the body outline of the animal	Related to capacity to avoid predators, by preventing prey recognition	Schaefer and Stobbe (2006)

Note

C—continuous traits and B—binary traits.

### 2.4 Incorporating imperfect detection in diversity measures: The hidden diversity framework

To evaluate the magnitude of the effects of imperfect detection on diversity measures, we developed an R function called *hidden.diversity* (HD) (Appendix S3—hidden diversity framework). This function returns, for each site *i*, the deviation of observed diversity from the estimated diversity, given imperfect detection, and this difference is divided by the standard deviation of the estimated diversity as follows:

$$\text{hidden}.{\text{diversity}}_{i}=\frac{\text{div}.{\text{obs}}_{i}‐\bar{\text{div}.{\text{est}}_{i}}}{\text{sd}.\text{div}.{\text{est}}_{i}}$$

where *div.obs* *_i_* is the taxonomic, functional, or phylogenetic diversity value obtained with observed count data for each site, $\bar{{\text{div}\text{.est}}_{i}}$ is the mean diversity value obtained from *N_ik_* posterior sampling in each site, and *sd.div.est* *_i_* is the standard deviation of *div.est* *_i_*. Positive and negative values of HD indicate, respectively, an overestimation and underestimation of observed diversity to estimated diversity values. Overestimation of diversity can only occur for phylogenetic or functional measures, since that species included can be functionally or phylogenetically redundant, and the N‐mixture model only accounts for false negatives. However, distinct scenarios can generate positive or negatives HD values, and we disentangle these possible scenarios by plotting the relationship between observed and estimated diversity values (Figure 1c). We called noise when observed and estimated diversity has the same sign; in other words, the observed pattern (overdispersion or clustering) does not change after corrected by imperfect detection, but still can be overestimated or underestimated in comparison with the estimated true diversity. On the other hand, a bias will occur if the observed and estimated diversity has opposite signs, and for these cases, an erroneous pattern in phylogenetic and/or functional structure of communities will be observed when undetected species are not considered.

The input of the *hidden.diversity* function is the observed community data, a phylogenetic tree, a matrix containing the mean traits for each species, and the matrix *N_ik_* estimated by the N‐mixture model which represents the detection‐corrected abundance. The function internally always calculates taxonomic diversity (TD) and abundance, and allows the user to choose which diversity metrics should be calculated—“*pd*” for branch length and “*mpd*” for distance‐based approach—for both phylogenetic and functional diversity. The function will calculate the standardized effect size for phylogenetic diversity (SES.PD) and functional diversity (SES.FD) if only “*pd*” is informed and the SES for phylogenetic and functional structure (SES.MPD and SES.MFD, respectively) if only “*mpd*” is informed, or both if the user wishes. Also, the function allows indicating if there are binary data in the trait matrix, if the diversity measures should be weighted by abundance, the type of null model, the number of permutations used to calculate the null models. Null models allow removing the effect of species richness on diversity measures by randomizing communities, permuting by permuting the positions of species in the phylogenetic tree or functional dendrograms, or by permuting the sampling units (rows) or species identities (columns) in the community matrix. Null models are implemented in the package *picante* (Kembel et al., 2010). The function output is a data frame containing SES values of diversity measures for each site (observed and estimated) and the value of hidden diversity.

We employed the HD for each diversity measure to evaluate differences between canopy and understory in the bias yielded by imperfect detection. For this, we performed a linear mixed model (LMM) using the HD values for each diversity measure as the response variable, the strata as a fixed predictor, and the sampling months and sites as random factors. We used the *lme4* package (v. 4.0.5, Bates et al., 2015) to perform the LMM and the *ggplot2* package (v. 3.3.4 Wickham, 2016) to graphical visualization of the results.

## 3 RESULTS

**FIGURE 2:**
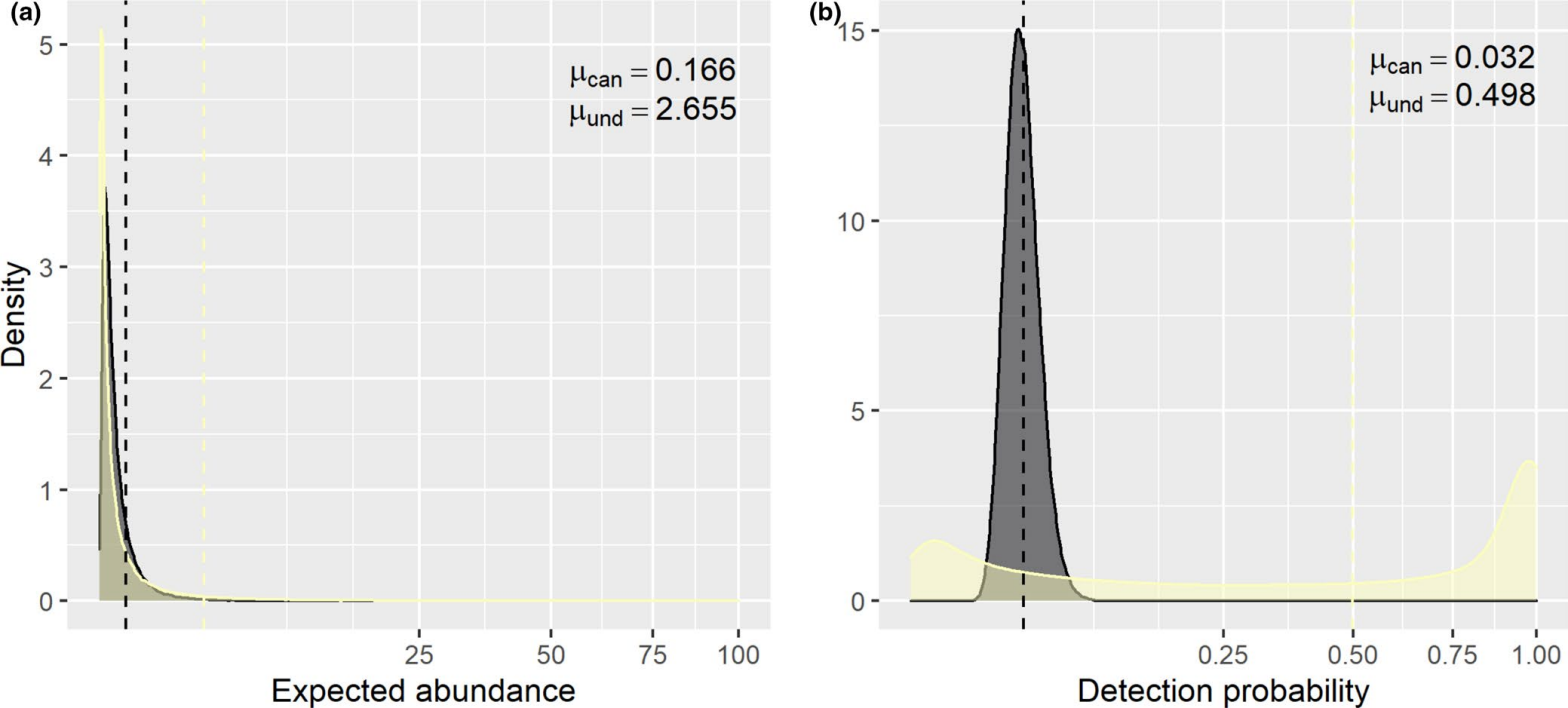
Community‐mean distribution for expected abundance (a) and detection probability (b) for fruit‐feeding butterflies sampled at FLONA‐SFP, southern Brazil. These distributions were generated using the community hyperparameters for canopy (*µ*_can_ and sd_can_, black color) and understory (*µ*_und_ and sd_und_, light yellow color). The dashed line represents the mean for each stratum (*µ*). We apply a square root transformation on the x‐axis to better improve the visualization

Our database contained 35 species and 914 individuals of fruit‐feeding butterflies. We found that canopy had lower community‐level mean abundance than understory (values in the natural scale, *µ_β_*_.can_ = 0.166 CRI_95%_ = 0.008 to 0.104, *µ_β_*_.und_ = 2.655, CRI_95%_ = 0.001 to 0.117). Moreover, understory assemblages had a higher mean detection probability (values in the natural scale, *µ_α_*_.can_ = 0.032, CRI_95%_ = 0.025 to 0.038, *µ_α_*_.und_ = 0.497, CRI_95%_ = 0.033 to 0.964) (Figure 2). We do not explore the effects of predictor variables on abundance and detection probability because these results are not crucial for this study, but the values for hyperparameters for community level are shown in Appendix S1 (Figures A1 and A2; Table A1).

**FIGURE 3:**
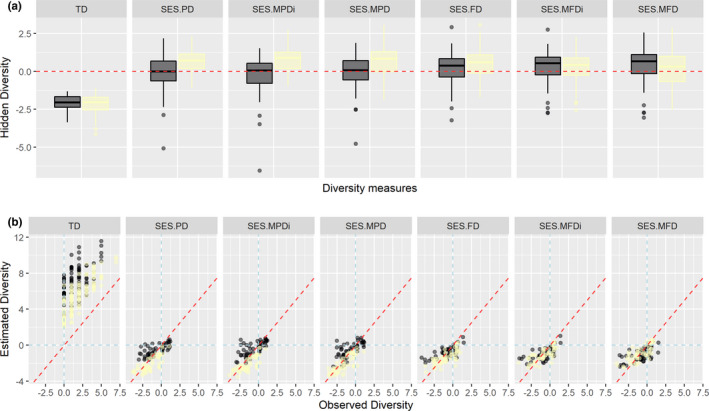
The effects of imperfect detection on multiple dimensions of biodiversity, evaluated by the hidden diversity framework for an assemblage of fruit‐feeding butterflies sampled at FLONA‐SFP, southern Brazil. (a) Response of each stratum—canopy (dark boxplots) and understory (light yellow boxplots)—to the imperfect detection and their variation among the diversity measures. TD—taxonomic diversity, SES—standardized effect size, PD/FD—phylogenetic/functional diversity, MPD/MFD—abundance‐based mean pairwise phylogenetic/functional distance, and MPDi/MFDi—incidence‐based mean pairwise phylogenetic/functional distance. The red dashed line indicates no difference in diversity value between observed and estimated data. (b) Visual evaluation of the effect of the imperfect detection by sampling unit (points) and environmental factors (colors, dark—canopy; yellow—understory). Points above the dashed red line indicate an underestimate of the diversity and negative values of hidden diversity; points below this line indicate an overestimation of diversity and positive values of hidden diversity

Hidden diversity (HD) demonstrated that there was an underestimation for both strata when only the species richness was evaluated (TD), and for this diversity measure, the HD differed between strata (Figure 3a; Table 2). All other diversity measures tended to be overestimated (positive HD values). Phylogenetic and functional measures had opposite responses concerning the most overestimated stratum: while for phylogenetic measures, understory was more overestimated than the canopy, for functional measures canopy tended to show higher overestimation than understory. Only for functional richness (SES.FD) we did not observe a difference in the HD between strata (Table 2). However, observing the relationship among observed and estimated diversity, we found that for most sites, the pattern of positive or negative SES value was maintained. This implies that, despite the error associated with not accounting for imperfect detection, for the fruit‐feeding butterfly assembly, imperfect detection acts more like a noise than a bias (Figure 3b).

**TABLE 2:** Relationship of hidden diversity values for taxonomic, phylogenetic, and functional measures (HD.TD, HD.PD/MPD, HD.FD.MFD) and vertical stratification for the assemblage of fruit‐feeding butterflies sampled at FLONA‐SFP, southern Brazil

	Estimate	*SE*	*t* value	*p*
HD.TD				
Intercept	−2.064	0.163	−12.679	.**000**
Slope	−0.099	0.046	−2.163	.**031**
HD.PD				
Intercept	0.174	0.090	1.931	.092
Slope	0.728	0.090	8.060	.**000**
HD.FD				
Intercept	0.509	0.064	7.955	.**000**
Slope	−0.032	0.090	−0.349	.727
HD.MPDi*				
Intercept	0.076	0.128	0.595	.572
Slope	0.938	0.095	9.849	.**000**
HD.MPD				
Intercept	0.226	0.177	1.279	.240
Slope	0.848	0.104	8.164	.**000**
HD.MFDi*				
Intercept	0.619	0.063	9.838	.**000**
Slope	−0.312	0.089	−3.511	.**001**
HD.MFD				
Intercept	0.680	0.094	7.212	.**000**
Slope	−0.564	0.101	−5.563	.**000**

Note

Bold values indicate a statistical significance at a threshold of 0.05. The asterisk indicates values of mean pairwise distances calculated with incidence instead of abundance.

## 4 DISCUSSION

Our results demonstrate that neglect imperfect detection can produce unrealistic estimates of diversity, which can be unbalanced between treatment levels or environmental gradients. Considering that several community studies are pattern‐based, ignoring the effect of imperfect detection can lead to spurious interpretations of the mechanisms driving community assembly (Joseph et al., 2009), mainly when inversion in the observed pattern occurs (critical bias regions, Figure 1c). For the assemblage of fruit‐feeding butterflies studied, we found a noise for site scale (the majority of points are in the noise region, Figure 3b), typically produced by the inclusion of redundant species at understory for phylogenetic measures and redundant species at the canopy for functional measures. This occurs because the capacity to detect distinct lineages or functional traits in both strata was higher than the ability to detect new species (Jarzyna & Jetz, 2016), leading to an increase in phylogenetic or functional clustering in relation to the observed data. However, since there is a difference in the detection of individuals between strata (reached by hidden diversity), the relationship between diversity and the environment is biased.

Canopy and understory have distinct features including microclimatic conditions, forest structure, and resource availability (Grimbacher & Stork, 2007; Sobek et al., 2009). Such differences are commonly associated with the vertical stratification of animal taxa, especially for insects (Ashton et al., 2016; Basset et al., 2015). For fruit‐feeding butterflies, is recognized that some families or tribes are associated with a particular vertical statum (DeVries et al., 2012; Hill et al., 2001), even the probability of species detection may differ between strata (Ribeiro et al., 2016). In addition to the lack of studies evaluating phylogenetic and functional diversity for this group, for the Neotropical region, there is no clear pattern as to which is the most diverse stratum from a taxonomic perspective (understory—Araujo et al., 2020; Barlow et al., 2007; Lourenço et al., 2019; Whitworth et al., 2016; canopy—Devries, 1988; DeVries et al., 2012; Ribeiro & Freitas, 2012; Santos et al., 2017). In our study, we show that there was a large underestimation in species richness, providing evidence that there is a bias for observed taxonomic diversity in canopies sites. This was the only case where there was an inversion in the observed pattern: Understory was richest than canopy employing the observed data, but the canopy has a higher richness than understory when we used the estimated data (Appendix S3—Figure C1; Table C1). For phylogenetic measures, despite the difference in HD values between stratum, the observed pattern was maintained and only the magnitude of the effect was adjusted. However, for functional measures based on distances (SES.MFD), the inclusion of undetected individuals revealed a significant difference (understory was more diverse than canopy), unobserved when only observed data were used (Appendix S3—Table C1).

As expected, the inclusion of undetected species had a larger effect on taxonomic diversity measures than on phylogenetic or functional ones. While for taxonomic diversity, each undetected species leads to an increment of the estimated diversity value, for phylogenetic and functional measures, undetected species may be redundant, that is, contain evolutionary or functional information, respectively, that was already covered in the observed data. Furthermore, we observed that the understory had a large number of species belonging to the same lineage that were undetected in the field. Generally, fruit‐feeding butterflies that inhabit the understory belong to Satyrinae (particularly to the tribes Morphini and Brassolini). These species tend to be more abundant during the summer months (December to February in Southern Hemisphere) (Iserhard et al., 2017), and hence, more individuals are available to be detected. But at the beginning or end of this season, a smaller number of individuals are active, hindering its detection. Such features could explain the clustered pattern observed in the understory when we include imperfect detection to perform phylogenetic measures. Similarly, a clustered pattern was revealed for functional measures for canopy. Species that occupy this stratum generally exhibit traits related to flight performance (Chai & Srygley, 1990; Graça et al., 2017), given high mobility to looking for resources and favorable conditions (Shahabuddin & Ponte, 2005). Thus, a simple explanation for the inclusion of redundant traits in the canopy could be that individuals were absent because they were visiting a part of their home area that was not covered by the survey (Joseph et al., 2009; Ribeiro et al., 2016). Future investigations should be conducted in this context to understand whether high flight mobility leads to a low probability of butterfly detection.

Biodiversity measures are important tools to guide species conservation decisions, as well as to infer about the ecological and evolutionary process that structure assemblages. Since accounting for imperfect detection improves the accuracy of estimates of diversity patterns, in some circumstances, it is strongly recommended (Figure 1), because it may lower the risk of erroneously inferring biological processes that are implied by sampling uncertainty (Joseph et al., 2009). Several models have been proposed in recent years to incorporate imperfect detection in order to improve the efficiency of estimating parameters in community studies (Abrams et al., 2021; Broms et al., 2015; Frishkoff et al., 2017; Jarzyna & Jetz, 2016; Tingley et al., 2020; Zipkin et al., 2010). Further, these models allow us to propagate the uncertainty in species‐specific detectability to biodiversity measures, as we demonstrated here. We expect that the framework developed in this study helps researchers to better understand and describe diversity patterns and the mechanisms that assemble ecological communities.

## CONFLICT OF INTEREST

None declared.

## AUTHOR CONTRIBUTIONS

**Aline Richter:** Conceptualization (lead); data curation (lead); formal analysis (lead); investigation (lead); methodology (lead); project administration (lead); validation (lead); visualization (lead); writing–original draft (lead); writing–review and editing (lead). **Gabriel Nakamura:** Conceptualization (supporting); formal analysis (supporting); methodology (supporting); validation (supporting); visualization (supporting); writing–review and editing (supporting). **Cristiano Agra Iserhard:** Conceptualization (supporting); data curation (supporting); funding acquisition (supporting); investigation (supporting); supervision (supporting); writing–review and editing (supporting). **Leandro da Silva Duarte:** Conceptualization (lead); formal analysis (supporting); funding acquisition (supporting); methodology (supporting); supervision (lead); writing–original draft (supporting); writing–review and editing (supporting).

### OPEN RESEARCH BADGES

This article has earned an Open Data Badge for making publicly available the digitally‐shareable data necessary to reproduce the reported results. The data is available at 10.5281/zenodo.4876275.

## Supporting information

Appendix S1

Appendix S2

Appendix S3

## Data Availability

The code and all necessary data to perform all analyses made in this manuscript, as well the code for the hidden diversity framework, are available on Zenodo:10.5281/zenodo.5132227.
