# Supplementary material for: The hidden side of diversity: Effects of imperfect detection on multiple dimensions of biodiversity
Source: Ecol Evol. 2021 Aug 10;11(18):12508–19. doi: 10.1002/ece3.7995 (PMC8462181; doi:10.1002/ece3.7995)
Supplement: Supplementary file 3 — Appendix S3 [file ECE3-11-12508-s002.docx]

**Appendix S3 - Hidden diversity framework**

Function to evaluate the extent to which imperfect detection may affect patterns of taxonomic, functional, and phylogenetic diversities in ecological communities This function allows the user to estimate, in the form of a Standardized Effect Size (SES), how much of the diversity was hidden when we do not account with the imperfect detection. Further, the user can calculate the hidden diversity only for taxonomic diversity (TD and Abundance), for functional (SES.FD and SES.MFD), and taxonomic diversity, for phylogenetic (SES.PD and SES.MPD) and taxonomic diversity, or for all measures.

**Arguments**

comm = Community data, with sites in the rows and species in the columns.

N = an array when each “slice” is a matrix of the true-abundance (sites in rows and species in columns) for one posterior sampling of the N-mixture model. This object represents the detection-corrected abundance.

phy = a phylogenetic tree, with branch length.

trait = a trait community matrix, with species in rows and traits in columns.

Metric = a string with one or two options c(“pd”, “mpd”). If only “pd” is provided, the function will calculate the SES (standardized effect size) of the sum of the branch length for each community. If only “mpd” is provided, the function will calculate the SES for the mean pairwise distance of species that compose each community. Default is c(“pd”, “mpd”). This argument only works if a phylogenetic tree or a trait matrix is provided.

binary = logical. Default is FALSE. Only necessary when a trait matrix is present. If binary = TRUE, the function is taken into account binary traits for construct a functional dendrogram.

abundance.weighted = logical. Default is FALSE. In this case, for SES.MPD and SES.MFD only the occurrence/incidence/richness of species is accounted for by calculating the diversity values. If abundance.weighted = TRUE, the SES.MPD and SES.MFD will be calculated for an abundance-based community matrix.

Null.model = a string with the null models allowed by ses.pd and ses.mpd function from *picante* package.

Runs = number of permutations used to calculate the null models.

Parallel = number of cores used to process the function

**Value**

The function returns a list with two to six data frames (dependent on imputed diversity):

TD, Abund = always returned. Each object is a data frame with four columns: the observed richness/abundance, the mean richness/abundance for estimated data (N), the standard deviation of the estimated richness/abundance, and the hidden diversity.

sesPD, sesFD, sesMPD and sesMFD = only returned if the user informed a phylogenetic tree and/or a functional traits matrix. Each object has four columns: the SES value for observed diversity, the SES values for mean estimated diversity, the standard deviation for estimated SES, and the hidden diversity.

hidden.diversity <- function(comm, N, phy = NULL, trait = NULL, metrics = c("pd", "mpd"), binary = FALSE, abundance.weighted = FALSE, null.model = "taxa.labels", runs = 499, parallel = 3) {

n.site <- dim(N)[1] # n.site: the number of sampling sites

n.samp <- dim(N)[3] # n.samp: the number of posterior sampling

# transforming N in occurrence data (y)

y <- N

for (i in 1: dim(y)[3]) {

b = which(y[,,i] > 0)

y[,,i][b] = 1

y[,,i][-b] = 0

}

# calculating the observed and estimated richness (TD)

TD.df <- data.frame(TD.obs = apply(vegan::decostand(x = comm, method = "pa"), 1, sum), TD.est = apply(apply(y, c(1,3), sum), 1, mean), TD.sd = apply(apply(y, c(1,3), sum), 1, sd))

TD.df$HD.TD <- (TD.df$TD.obs - TD.df$TD.est)/TD.df$TD.sd

# calculating the observed and estimated abundance

N.df <- data.frame(N.obs = apply(comm, 1, sum), N.est = apply(apply(N, c(1,3), sum), 1, mean), N.sd = apply(apply(N, c(1,3), sum), 1, sd))

N.df$HD.N <- (N.df$N.obs - N.df$N.est) / N.df$N.sd

div_measures <- c("rich", "abund", "pd", "mpd")

hd_metric <- pmatch(metrics, div_measures)

# if a phylogenetic tree is provided

if (!is.null(phy)){

if(any(hd_metric == 3)){ # calculation of PD

# observed data

pd.obs <- picante::ses.pd(samp = comm, tree = phy, null.model = null.model, runs = runs, include.root = F)

# estimated data

if (is.numeric(parallel)) {

CL1 <- parallel::makeCluster(parallel, type = "PSOCK")

newClusters <- TRUE

}

if (!inherits(CL1, "cluster")) {

pd.ses <- array(NA, dim = c(n.site, 2, n.samp))

for (i in 1:n.samp){

temp_pd <- picante::ses.pd(samp = N[,,i], tree = phy, null.model = null.model, runs = runs, include.root = F)

pd.ses[ , 1, i] <- cbind(temp_pd[,6])

}

PD.df <- data.frame(SES.PD.obs = pd.obs[ ,"pd.obs.z"], SES.PD.est = apply(pd.ses[,1,], 1, mean, na.rm = T), SES.PD.sd = apply(pd.ses[,1,], 1, sd, na.rm = T))

}

else {

res_sesPD_samp <- parallel::parApply(cl = CL1, MARGIN = 3, X = y, FUN = picante::ses.pd, tree = phy, null.model = null.model, include.root = F, runs = runs)

HD.comm <- list(SES.PDest = res_sesPD_samp)

PD_est <- matrix(unlist(lapply(HD.comm$SES.PDest, function(x) x$pd.obs.z)), nrow = nrow(comm), ncol = dim(y)[3], dimnames = list(rownames(comm), paste("samp", 1:dim(y)[3], sep = "_")))

matrix_mean_SES_PD <- data.frame(matrix(c(apply(PD_est, MARGIN = 1, mean), apply(PD_est, MARGIN = 1, sd)), nrow = nrow(comm), ncol = 2, dimnames = list(rownames(comm), c("mean_ses.pd", "sd_ses.pd")), byrow = FALSE))

PD.df <- data.frame(SES.PD.obs = pd.obs$pd.obs.z, SES.PD.est = matrix_mean_SES_PD$mean_ses.pd, SES.PD.sd = matrix_mean_SES_PD$sd_ses.pd)

}

# calculating the Hidden Diversity for SES.PD

PD.df$HD.PD <- (PD.df$SES.PD.obs - PD.df$SES.PD.est) / PD.df$SES.PD.sd

}

if(any(hd_metric == 4)){ # calculation of MPD

# observed data

mpd.obs <- picante::ses.mpd(samp = comm, dis = cophenetic(x = phy), null.model = null.model, runs = runs)

# estimated data

mpd.ses <- array(NA, dim = c(n.site, 2, n.samp))

if (is.numeric(parallel)) {

CL1 <- parallel::makeCluster(parallel, type = "PSOCK")

newClusters <- TRUE

}

if (!inherits(CL1, "cluster")) {

mpd.ses <- array(NA, dim = c(n.site, 1, n.samp))

for (i in 1:n.samp){

temp_mpd <- picante::ses.mpd(samp = N[,,i], dis = cophenetic(x = phy), null.model = null.model, runs = runs)

mpd.ses[,1,i] <- cbind(temp_mpd[,6])

}

MPD.df <- data.frame(SES.MPD.obs = mpd.obs[ , "mpd.obs.z"], SES.MPD.est = apply(mpd.ses[,1,], 1, mean, na.rm = T), SES.MPD.sd = apply(mpd.ses[,1,], 1, sd, na.rm = T))

}

else {

res_sesMPD_samp <- parallel::parApply(cl = CL1, MARGIN = 3, X = y, FUN = picante::ses.mpd, dis = cophenetic(phy), abundance.weighted = abundance.weighted, null.model = null.model, runs = runs)

HD.comm <- list(SES.MPDest = res_sesMPD_samp)

MPD_est <- matrix(unlist(lapply(HD.comm$SES.MPDest, function(x) x$mpd.obs.z)), nrow = nrow(comm), ncol = dim(y)[3], dimnames = list(rownames(comm), paste("samp", 1:dim(y)[3], sep = "_")))

matrix_mean_SES_MPD <- data.frame(matrix(c(apply(MPD_est, MARGIN = 1, mean), apply(MPD_est, MARGIN = 1, sd)), nrow = nrow(comm), ncol = 2, dimnames = list(rownames(comm), c("mean_ses.pd", "sd_ses.mpd")), byrow = FALSE))

MPD.df <- data.frame(SES.MPD.obs = mpd.obs$mpd.obs.z, SES.MPD.est = matrix_mean_SES_MPD$mean_ses.pd, SES.MPD.sd = matrix_mean_SES_MPD$sd_ses.mpd)

}

# hidden for mpd phylo

MPD.df$HD.MPD <- (MPD.df$SES.MPD.obs - MPD.df$SES.MPD.est) / MPD.df$SES.MPD.sd

}

}

# calculation for traits

if (!is.null(trait)){

if(binary == TRUE){

bin <- vector()

for(i in 1:ncol(trait)){

bin[i] <- is.integer(trait[, i]) | is.factor(trait[, i])

}

con.t <- which(bin == F)

bin.t <- which(bin == T)

t.dist <- ade4::dist.ktab(ade4::ktab.list.df(list(log(trait[, con.t]), ade4::prep.binary(trait[, bin.t], col.blocks = ncol(trait[, bin.t])))), type = c("Q", "B")) # create a dist matrix, considering mixed-variables

}

else {

t.dist <- ade4::dist.ktab(ade4::ktab.list.df(list(log(trait))), type = "Q")

}

tree.func <- hclust(d = t.dist, method = "average") # clustering using UPGMA

tree.func <- ape::as.phylo(tree.func)

if(any(hd_metric == 3)){ # calculation of FD

# observed data

fd.obs <- picante::ses.pd(samp = comm, tree = tree.func, null.model = null.model, runs = runs, include.root = F)

# estimated data

if (is.numeric(parallel)) {

CL1 <- parallel::makeCluster(parallel, type = "PSOCK")

newClusters <- TRUE

}

if (!inherits(CL1, "cluster")) {

fd.ses <- array(NA, dim = c(n.site, 1, n.samp))

for (i in 1:n.samp){

temp_fd <- picante::ses.pd(samp = N[,,i], tree = tree.func, null.model = null.model, runs = runs, include.root = F)

fd.ses[ , 1, i] <- cbind(temp_fd[,6])

}

FD.df <- data.frame(SES.FD.obs = fd.obs[ , "pd.obs.z"],SES.FD.est = apply(fd.ses[,1,], 1, mean, na.rm = T), SES.FD.sd = apply(fd.ses[,1,], 1, sd, na.rm = T))

}

else {

res_sesFD_samp <- parallel::parApply(cl = CL1, MARGIN = 3, X = y, FUN = picante::ses.pd, tree = tree.func, null.model = null.model, include.root = F, runs = runs)

HD.comm <- list(SES.FDest = res_sesFD_samp)

FD_est <- matrix(unlist(lapply(HD.comm$SES.FDest, function(x) x$pd.obs.z)), nrow = nrow(comm), ncol = dim(y)[3], dimnames = list(rownames(comm), paste("samp", 1:dim(y)[3], sep = "_")))

matrix_mean_SES_FD <- data.frame(matrix(c(apply(FD_est, MARGIN = 1, mean), apply(FD_est, MARGIN = 1, sd)), nrow = nrow(comm), ncol = 2, dimnames = list(rownames(comm), c("mean_ses.fd", "sd_ses.fd")), byrow = FALSE))

FD.df <- data.frame(SES.FD.obs = fd.obs$pd.obs.z, SES.FD.est = matrix_mean_SES_FD$mean_ses.fd, SES.FD.sd = matrix_mean_SES_FD$sd_ses.fd)

}

# hidden FD

FD.df$HD.FD <- (FD.df$SES.FD.obs - FD.df$SES.FD.est) / FD.df$SES.FD.sd

}

if(any(hd_metric == 4)){

dist.func <- cophenetic(x = tree.func)

mfd.obs <- picante::ses.mpd(samp = comm, dis = dist.func, null.model = null.model, runs = runs)

mfd.ses <- array(NA, dim = c(n.site, 1, n.samp))

if (is.numeric(parallel)) {

CL1 <- parallel::makeCluster(parallel, type = "PSOCK")

newClusters <- TRUE

}

if (!inherits(CL1, "cluster")) {

for (i in 1:n.samp){

temp_mfd <- picante::ses.mpd(samp = N[,,i], dis = dist.func, null.model = null.model, runs = runs)

mfd.ses[ , 1, i] <- cbind(temp_mfd[, 6])

}

MFD.df <- data.frame(SES.MFD.obs = mfd.obs[ , "mpd.obs.z"], SES.MFD.est = apply(mfd.ses[,1,], 1, mean, na.rm = T), SES.MFD.sd = apply(mfd.ses[,1,], 1, sd, na.rm = T))

}

else {

res_sesMFD_samp <- parallel::parApply(cl = CL1, MARGIN = 3, X = y, FUN = picante::ses.mpd, dis = cophenetic(tree.func), abundance.weighted = abundance.weighted, null.model = null.model, runs = runs)

HD.comm <- list(SES.MFDest = res_sesMFD_samp)

MFD_est <- data.frame(matrix(unlist(lapply(HD.comm$SES.MFDest, function(x) x$mpd.obs.z)), nrow = nrow(comm), ncol = dim(y)[3], dimnames = list(rownames(comm), paste("samp", 1:dim(y)[3], sep = "_"))))

matrix_mean_SES_MFD <- data.frame(matrix(c(apply(MFD_est, MARGIN = 1, mean), apply(MFD_est, MARGIN = 1, sd)), nrow = nrow(comm), ncol = 2, dimnames = list(rownames(comm), c("mean_ses.mfd", "sd_ses.mfd")), byrow = FALSE))

MFD.df <- data.frame(SES.MFD.obs = mfd.obs$mpd.obs.z, SES.MFD.est = matrix_mean_SES_MFD$mean_ses.mfd, SES.MFD.sd = matrix_mean_SES_MFD$sd_ses.mfd)

}

# hidden mfd

MFD.df$HD.MFD <- (MFD.df$SES.MFD.obs - MFD.df$SES.MFD.est) / MFD.df$SES.MFD.sd

}

}

if (newClusters) {

parallel::stopCluster(CL1)

}

if(!is.null(trait) & !is.null(phy)){

if(all(hd_metric == c(3, 4))){

list_res <- vector(mode = "list", length = 6)

names(list_res) <- c("TD", "Abund", "sesPD", "sesMPD", "sesFD", "sesMFD")

list_res$TD <- TD.df

list_res$Abund <- N.df

list_res$sesPD <- PD.df

list_res$sesMPD <- MPD.df

list_res$sesFD <- FD.df

list_res$sesMFD <- MFD.df

for (i in 3:length(list_res)) {

pos_obs_na <- which(is.na(list_res[[i]][,1]) == TRUE & is.na(list_res[[i]][,2]) == FALSE)

list_res[[i]][pos_obs_na, paste("HD", gsub("ses","", names(list_res)[i]), sep = ".")] <- list_res[[i]][pos_obs_na, 2]/list_res[[i]][pos_obs_na, 3]

}

return(list_res)

}

else {

if(hd_metric == 3){

list_res <- vector(mode = "list", length = 4)

names(list_res) <- c("TD", "Abund", "sesPD", "sesFD")

list_res$TD <- TD.df

list_res$Abund <- N.df

list_res$sesPD <- PD.df

list_res$sesFD <- FD.df

for (i in 3:length(list_res)) {

pos_obs_na <- which(is.na(list_res[[i]][,1]) == TRUE & is.na(list_res[[i]][,2]) == FALSE)

list_res[[i]][pos_obs_na, paste("HD", gsub("ses","", names(list_res)[i]), sep = ".")] <- list_res[[i]][pos_obs_na, 2]/list_res[[i]][pos_obs_na, 3]

}

return(list_res)

}

if(hd_metric == 4){

list_res <- vector(mode = "list", length = 4)

names(list_res) <- c("TD", "Abund", "sesMPD", "sesMFD")

list_res$TD <- TD.df

list_res$Abund <- N.df

list_res$sesMPD <- MPD.df

list_res$sesMFD <- MFD.df

for (i in 3:length(list_res)) {

pos_obs_na <- which(is.na(list_res[[i]][,1]) == TRUE & is.na(list_res[[i]][,2]) == FALSE)

list_res[[i]][pos_obs_na, paste("HD", gsub("ses","", names(list_res)[i]), sep = ".")] <- list_res[[i]][pos_obs_na, 2]/list_res[[i]][pos_obs_na, 3]

}

return(list_res)

}

}

}

if(is.null(phy) & !is.null(trait)){

if(all(hd_metric == c(3, 4))){

list_res <- vector(mode = "list", length = 4)

names(list_res) <- c("TD", "Abund", "sesFD", "sesMFD")

list_res$TD <- TD.df

list_res$Abund <- N.df

list_res$sesFD <- FD.df

list_res$sesMFD <- MFD.df

for (i in 3:length(list_res)) {

pos_obs_na <- which(is.na(list_res[[i]][,1]) == TRUE & is.na(list_res[[i]][,2]) == FALSE)

list_res[[i]][pos_obs_na, paste("HD", gsub("ses","", names(list_res)[i]), sep = ".")] <- list_res[[i]][pos_obs_na, 2]/list_res[[i]][pos_obs_na, 3]

}

return(list_res)

}

else{

if(any(hd_metric == 3)){

list_res <- vector(mode = "list", length = 3)

names(list_res) <- c("TD", "Abund", "sesFD")

list_res$TD <- TD.df

list_res$Abund <- N.df

list_res$sesFD <- FD.df

for (i in 3:length(list_res)) {

pos_obs_na <- which(is.na(list_res[[i]][,1]) == TRUE & is.na(list_res[[i]][,2]) == FALSE)

list_res[[i]][pos_obs_na, paste("HD", gsub("ses","", names(list_res)[i]), sep = ".")] <- list_res[[i]][pos_obs_na, 2]/list_res[[i]][pos_obs_na, 3]

}

return(list_res)

}

if(any(hd_metric == 4)){

list_res <- vector(mode = "list", length = 3)

names(list_res) <- c("TD", "Abund", "sesMFD")

list_res$TD <- TD.df

list_res$Abund <- N.df

list_res$sesMFD <- MFD.df

for (i in 3:length(list_res)) {

pos_obs_na <- which(is.na(list_res[[i]][,1]) == TRUE & is.na(list_res[[i]][,2]) == FALSE)

list_res[[i]][pos_obs_na, paste("HD", gsub("ses","", names(list_res)[i]), sep = ".")] <- list_res[[i]][pos_obs_na, 2]/list_res[[i]][pos_obs_na, 3]

}

return(list_res)

}

}

}

if(!is.null(phy) & is.null(trait)){

if(all(hd_metric == c(3, 4))){

list_res <- vector(mode = "list", length = 4)

names(list_res) <- c("TD", "Abund", "sesPD", "sesMPD")

list_res$TD <- TD.df

list_res$Abund <- N.df

list_res$sesPD <- PD.df

list_res$sesMPD <- MPD.df

for (i in 3:length(list_res)) {

pos_obs_na <- which(is.na(list_res[[i]][,1]) == TRUE & is.na(list_res[[i]][,2]) == FALSE)

list_res[[i]][pos_obs_na, paste("HD", gsub("ses","", names(list_res)[i]), sep = ".")] <- list_res[[i]][pos_obs_na, 2]/list_res[[i]][pos_obs_na, 3]

}

return(list_res)

}

else {

if(hd_metric == 3){

list_res <- vector(mode = "list", length = 3)

names(list_res) <- c("TD", "Abund", "sesPD")

list_res$TD <- TD.df

list_res$Abund <- N.df

list_res$sesPD <- PD.df

for (i in 3:length(list_res)) {

pos_obs_na <- which(is.na(list_res[[i]][,1]) == TRUE & is.na(list_res[[i]][,2]) == FALSE)

list_res[[i]][pos_obs_na, paste("HD", gsub("ses","", names(list_res)[i]), sep = ".")] <- list_res[[i]][pos_obs_na, 2]/list_res[[i]][pos_obs_na, 3]

}

return(list_res)

}

if(hd_metric == 4){

list_res <- vector(mode = "list", length = 3)

names(list_res) <- c("TD", "Abund", "sesMPD")

list_res$TD <- TD.df

list_res$Abund <- N.df

list_res$sesMPD <- MPD.df

for (i in 3:length(list_res)) {

pos_obs_na <- which(is.na(list_res[[i]][,1]) == TRUE & is.na(list_res[[i]][,2]) == FALSE)

list_res[[i]][pos_obs_na, paste("HD", gsub("ses","", names(list_res)[i]), sep = ".")] <- list_res[[i]][pos_obs_na, 2]/list_res[[i]][pos_obs_na, 3]

}

return(list_res)

}

}

}

}


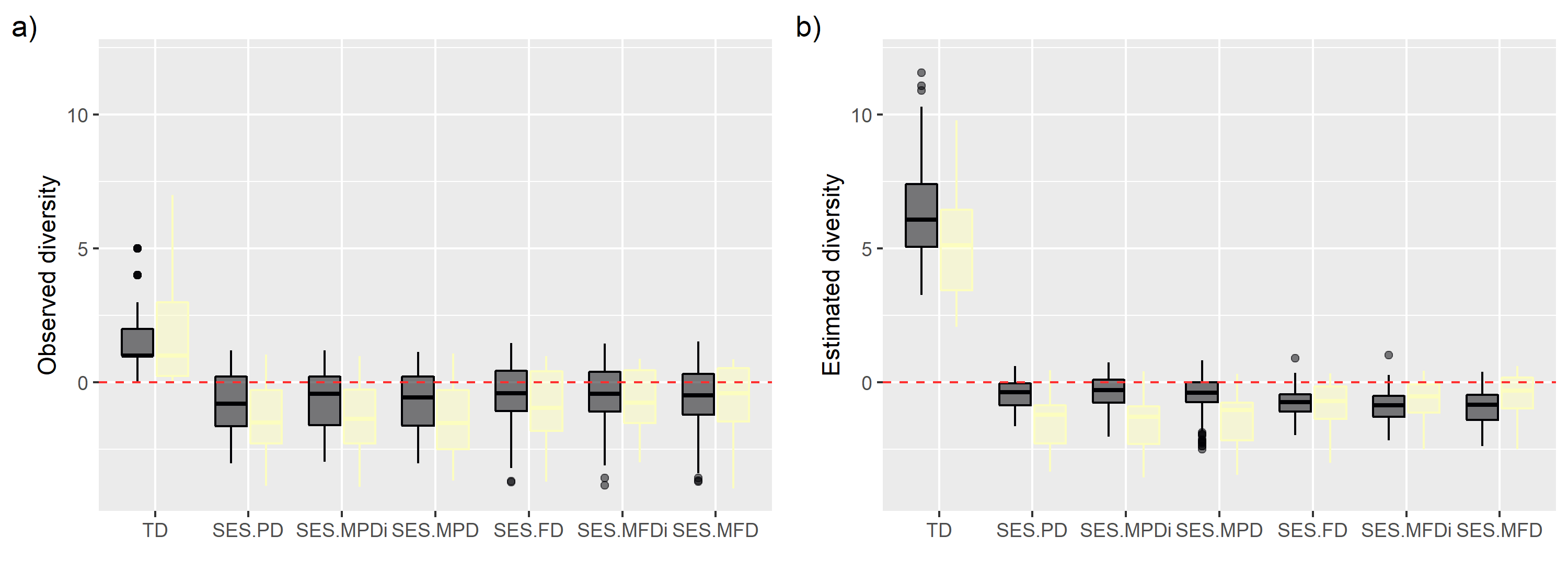


**Figure C1**. Relationship among diversity measures and canopy (dark boxplots) and understory (light yellow boxplots). a) Diversity pattern calculated using only observed data. b) Diversity pattern calculated using 100 matrices of estimated true abundance. TD – taxonomic diversity, SES – standardized effect size, PD/FD – phylogenetic/functional diversity, MPD/MFD – abundance-based mean pairwise phylogenetic/functional distance, MPDi/MFDi – incidence-based mean pairwise phylogenetic/functional distance.

**Table C1**. Relationship between distinct facets of biodiversity and vertical stratification for fruit-feeding butterflies community sampled at FLONA-SFP, southern Brazil. The first four columns show the relationship of diversity measures obtained by observed data with canopy and understory, and the last four columns show the relationship of diversity measures obtained by estimated data (corrected-by-detection). Bold values indicate a statistical significance at a threshold of 0.05. Asterisk indicates the unique case where there was an inversion of the most diverse stratum. TD – taxonomic diversity, SES – standardized effect size, PD/FD – phylogenetic/functional diversity, MPD/MFD – abundance-based mean pairwise phylogenetic/functional distance, MPDi/MFDi – incidence-based mean pairwise phylogenetic/functional distance.

|  | Observed data | | | |  | Estimated data | | | |
| --- | --- | --- | --- | --- | --- | --- | --- | --- | --- |
|  | Estimate | SE | t value | p |  | Estimate | SE | t value | p |
| TD |  |  |  |  |  |  |  |  |  |
| Canopy | 1.440 | 0.373 | 3.860 | **0.004** |  | 6.296 | 0.785 | 8.021 | **0.000** |
| Understory* | 0.280 | 0.140 | 2.003 | **0.046** |  | -1.169 | 0.088 | -13.35 | **0.000** |
| SES.PD |  |  |  |  |  |  |  |  |  |
| Canopy | -0.547 | 0.297 | -1.841 | 0.108 |  | -0.459 | 0.264 | -1.739 | 0.132 |
| Understory | -0.584 | 0.194 | -3.003 | **0.003** |  | -1.017 | 0.048 | -21.33 | **0.000** |
| SES.FD |  |  |  |  |  |  |  |  |  |
| Canopy | -0.589 | 0.315 | -1.873 | 0.094 |  | -0.772 | 0.213 | -3.619 | **0.009** |
| Understory | 0.009 | 0.200 | 0.047 | 0.963 |  | -0.038 | 0.049 | -0.764 | 0.446 |
| SES.MPDi |  |  |  |  |  |  |  |  |  |
| Canopy | -0.506 | 0.304 | -1.668 | 0.137 |  | -0.382 | 0.277 | -1.379 | 0.212 |
| Understory | -0.580 | 0.186 | -3.118 | **0.002** |  | -1.140 | 0.051 | -22.29 | **0.000** |
| SES.MPD |  |  |  |  |  |  |  |  |  |
| Canopy | -0.546 | 0.318 | -1.719 | 0.128 |  | -0.480 | 0.315 | -1.523 | 0.172 |
| Understory | -0.568 | 0.184 | -3.084 | **0.003** |  | -0.930 | 0.050 | -18.48 | **0.000** |
| SES.MFDi |  |  |  |  |  |  |  |  |  |
| Canopy | -0.626 | 0.306 | -2.044 | 0.072 |  | -0.914 | 0.218 | -4.199 | **0.004** |
| Understory | 0.087 | 0.201 | 0.433 | 0.666 |  | 0.276 | 0.045 | 6.188 | **0.000** |
| SES.MFD |  |  |  |  |  |  |  |  |  |
| Canopy | -0.680 | 0.341 | -1.996 | 0.076 |  | -0.922 | 0.263 | -3.505 | **0.008** |
| Understory | 0.175 | 0.202 | 0.866 | 0.388 |  | 0.463 | 0.044 | 10.61 | **0.000** |
